# Idiopathic Duodenocolic Fistula Presenting as Chronic Diarrhea: Successful Surgical Management After Failed Endoscopic Closure

**DOI:** 10.7759/cureus.99833

**Published:** 2025-12-22

**Authors:** Bruno Vieira, Ana Monteiro, João Pinto-de-Sousa

**Affiliations:** 1 General Surgery, Unidade Local de Saúde de Trás-os-Montes e Alto Douro, Vila Real, PRT; 2 Surgery, Clinical Academic Centre, Trás-os-Montes e Alto Douro, Vila Real, PRT

**Keywords:** chronic diarrhea, duodenocolic fistula, endoscopic clip failure, malabsorption, nutritional optimization, surgical management, weight loss

## Abstract

Idiopathic duodenocolic fistula is an exceptionally rare cause of chronic diarrhea, malabsorption, and weight loss. Management is challenging, especially after failed endoscopic closure. A 47-year-old man with no significant medical history presented with chronic diarrhea, cachexia (BMI 16.7 kg/m²), moderate anemia, and severe hypoalbuminemia. Radiography and MR enterography revealed a fistula between the second and third portions of the duodenum and the hepatic flexure of the colon. Two endoscopic over-the-scope clips (OTSC) closure attempts were unsuccessful. The patient underwent preoperative nutritional optimization, followed by surgical primary closure using a linear stapler. Histopathology confirmed benign chronic inflammation. Postoperatively, the patient achieved full recovery, with resolution of diarrhea and normalization of laboratory parameters at one-year follow-up. Although rare, an idiopathic duodenocolic fistula should be considered in patients with chronic diarrhea and malabsorption. This case underscores that in the absence of common etiologies and after failed endoscopic intervention, timely surgical management represents a highly effective and definitive curative strategy for idiopathic duodenocolic fistula.

## Introduction

Duodenocolic fistulas are rare abnormal communications between the duodenum and the colon, most frequently arising secondary to inflammatory bowel disease, malignancy, or prior abdominal surgery [1-3]. These fistulas typically present with chronic diarrhea, malnutrition, and weight loss due to rapid intestinal transit and nutrient malabsorption [2]. Idiopathic cases are exceptionally uncommon and remain scarcely reported in the literature. Their nonspecific presentation often delays diagnosis, making multimodality imaging essential for accurate characterization of the fistulous tract and exclusion of secondary causes [4-6]. Endoscopic closure, particularly with over-the-scope clips (OTSC), has emerged as a minimally invasive therapeutic option. Although encouraging results have been reported, success is heavily influenced by fistula chronicity, tissue fibrosis, and underlying etiology [7,8]. Surgical repair remains the most reliable definitive treatment when endoscopic management fails or when fistulas are large, epithelialized, or associated with significant inflammation [3,7]. This report describes a rare idiopathic duodenocolic fistula refractory to two OTSC closure attempts and successfully treated with surgical repair, highlighting key diagnostic considerations, definitive surgical management, and long-term outcome.

## Case presentation

A 47-year-old man, functionally independent and with no relevant medical history or medications, presented with chronic diarrhea, progressive weight loss, cachexia (BMI 16.7 kg/m²), moderate anemia (Hb 9.9 g/dL; ref: 13.5-17.5 g/dL), and severe hypoalbuminemia (2.2 g/dL; ref: 3.5-5.5 g/dL). He reported three to four diarrheic stools per day for several years, associated with significant impairment of quality of life, although appetite remained preserved.

**Figure 1:**
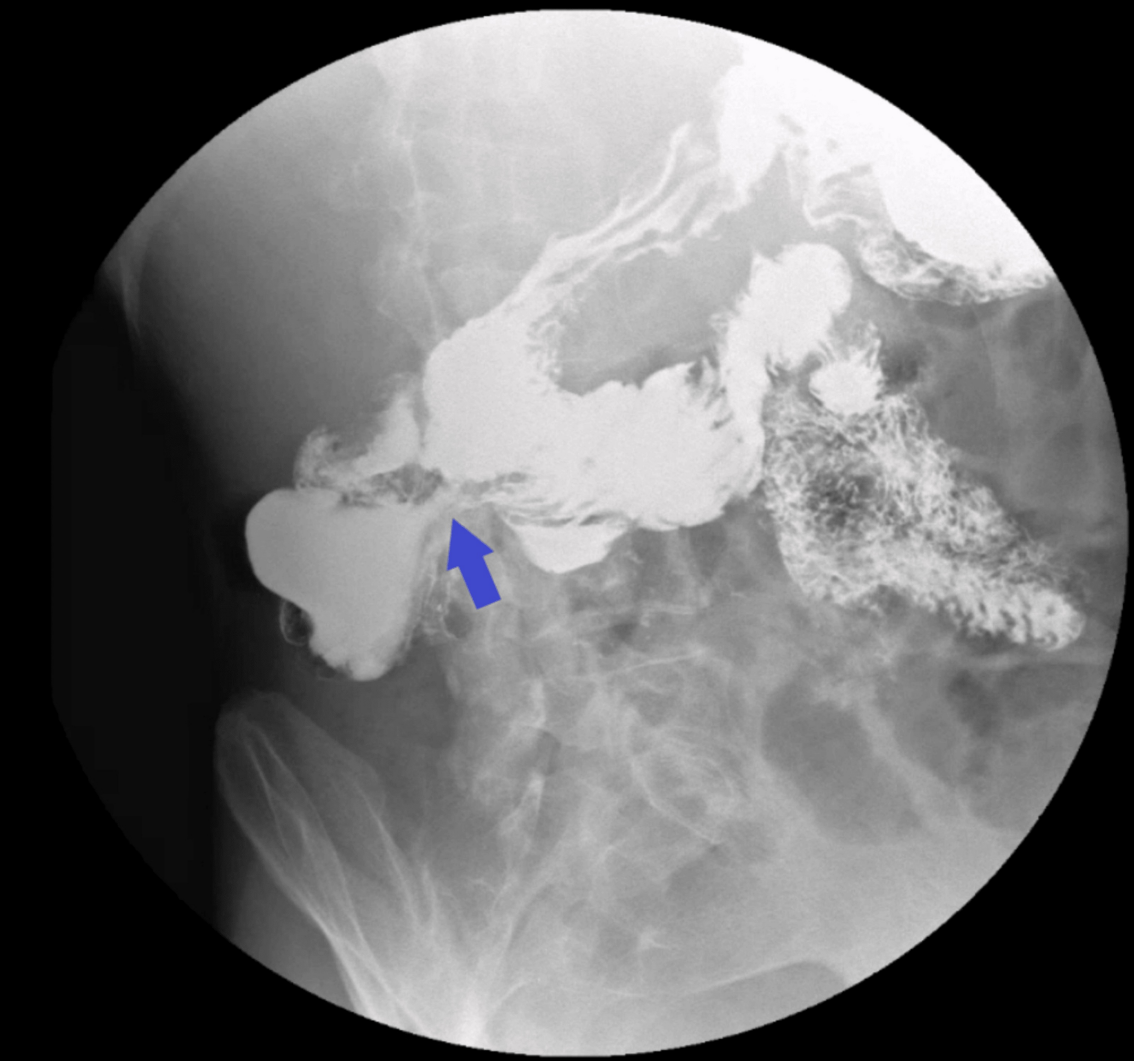
Early Contrast Passage From the Duodenum to the Colon Water-soluble contrast radiograph showing early and abnormal passage of contrast from the second portion of the duodenum to the hepatic flexure of the colon (blue arrow), confirming the presence of a duodenocolic fistula.

**Figure 2:**
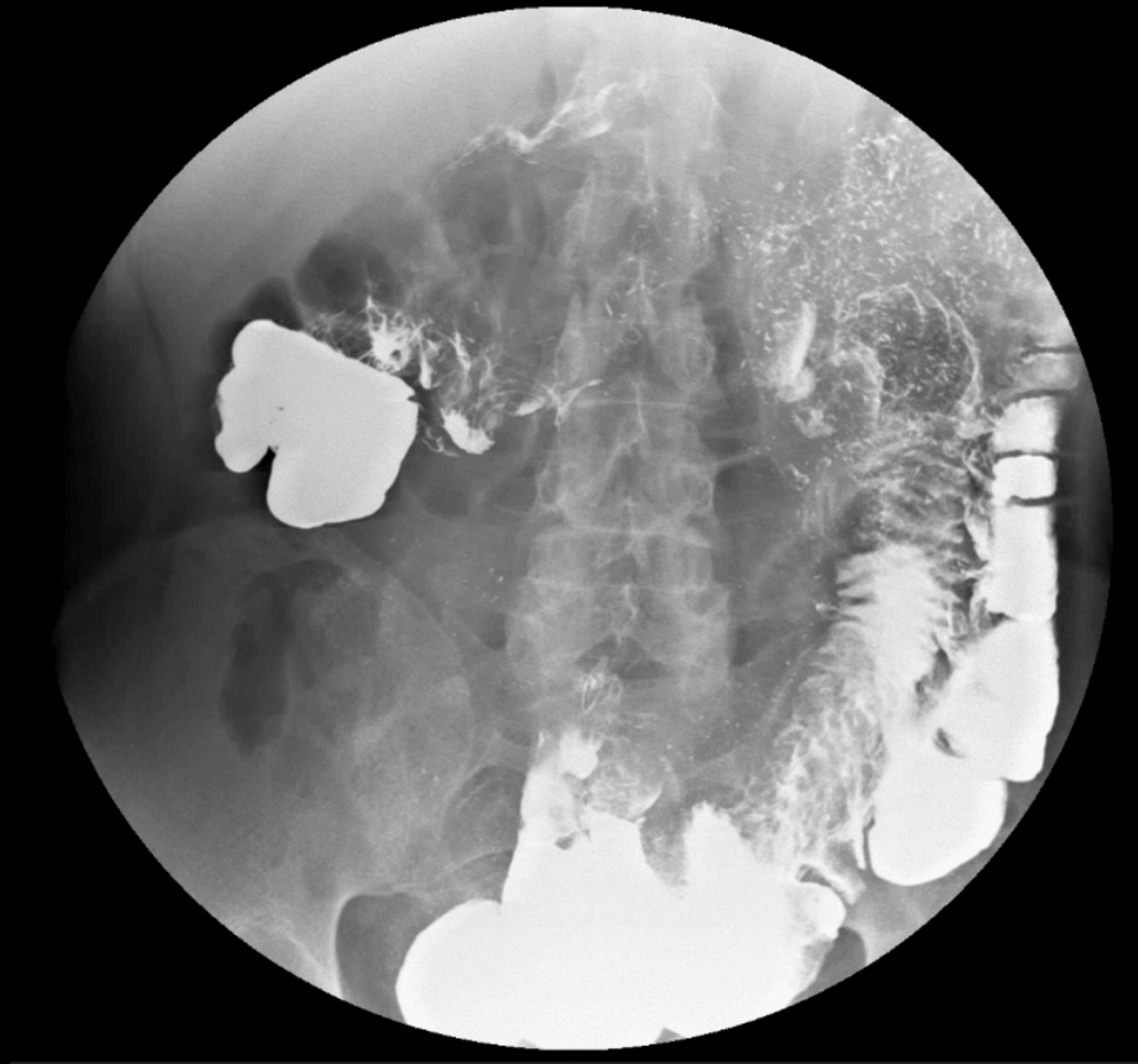
Accelerated Contrast Transit to the Distal Colon Water-soluble contrast radiograph demonstrating a rapid progression of contrast into the distal colon.

A water-soluble contrast study demonstrated early abnormal passage of contrast from the second portion of the duodenum to the hepatic flexure of the colon, establishing the diagnosis of a duodenocolic fistula (Figures 1, 2).

**Figure 3:**
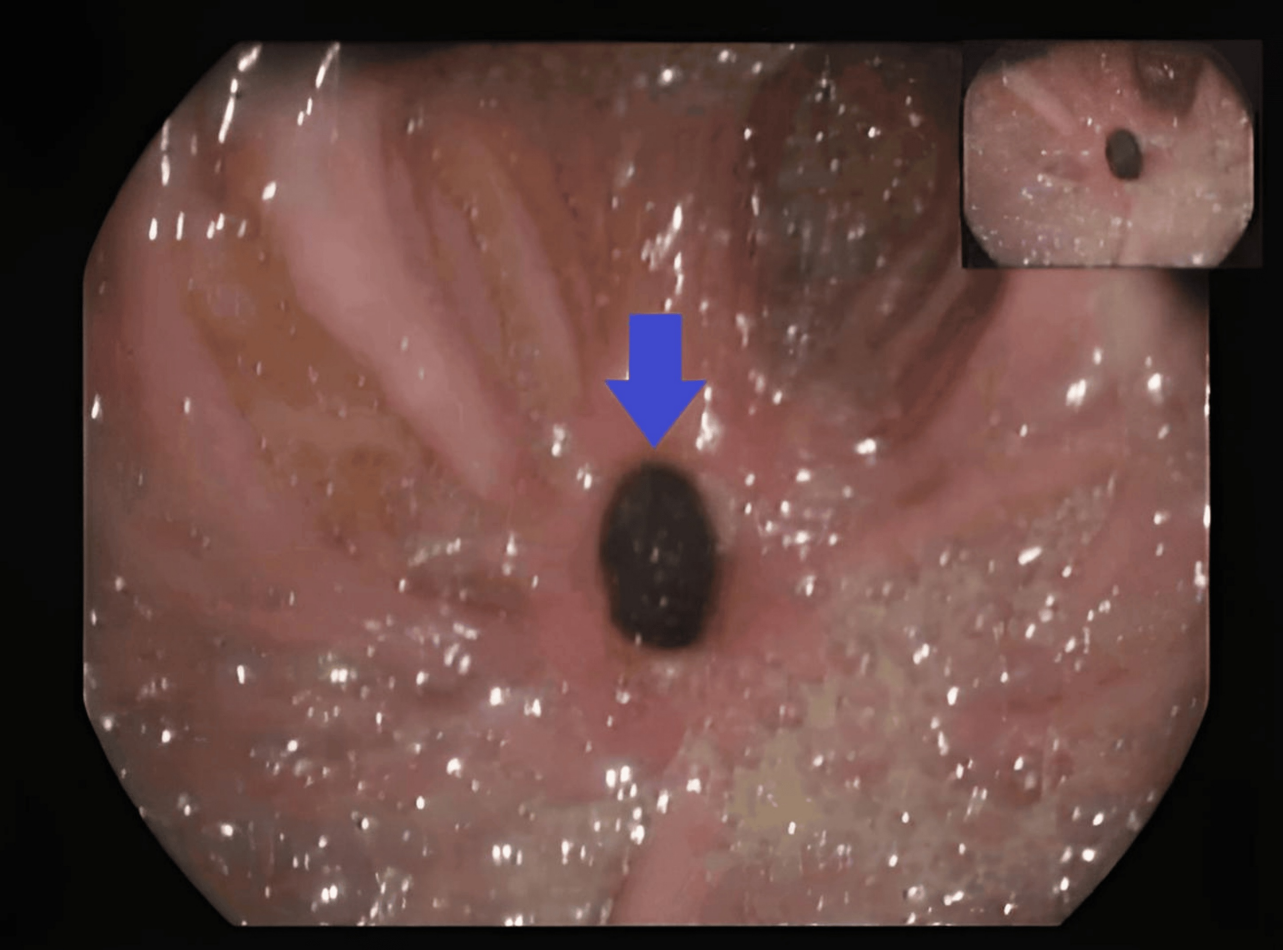
Endoscopic View of the Duodenocolic Fistula Orifice Endoscopic (duodenal) image showing the fistulous orifice of the duodenocolic fistula (blue arrow). Minor digital enhancement applied.

Upper endoscopy identified a 1-cm fistulous orifice at the transition between the second and third portions of the duodenum communicating with the hepatic flexure (Figure 3).

**Figure 4:**
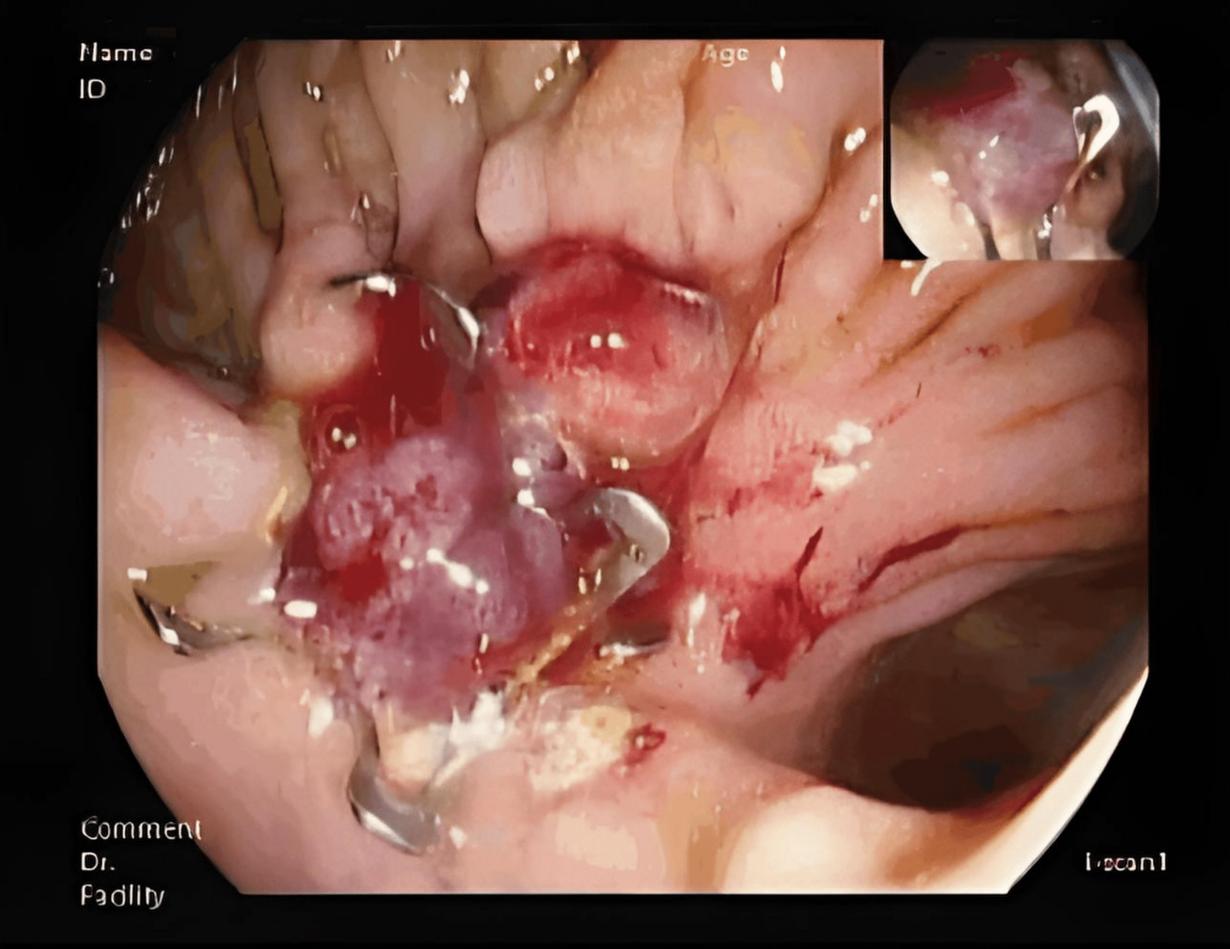
Endoscopic Closure Attempt Using an OTSC Upper endoscopy (duodenal view) after attempted closure of the fistula with an over-the-scope clip (OTSC) positioned over the fistulous orifice. Minor digital enhancement applied.

**Figure 5:**
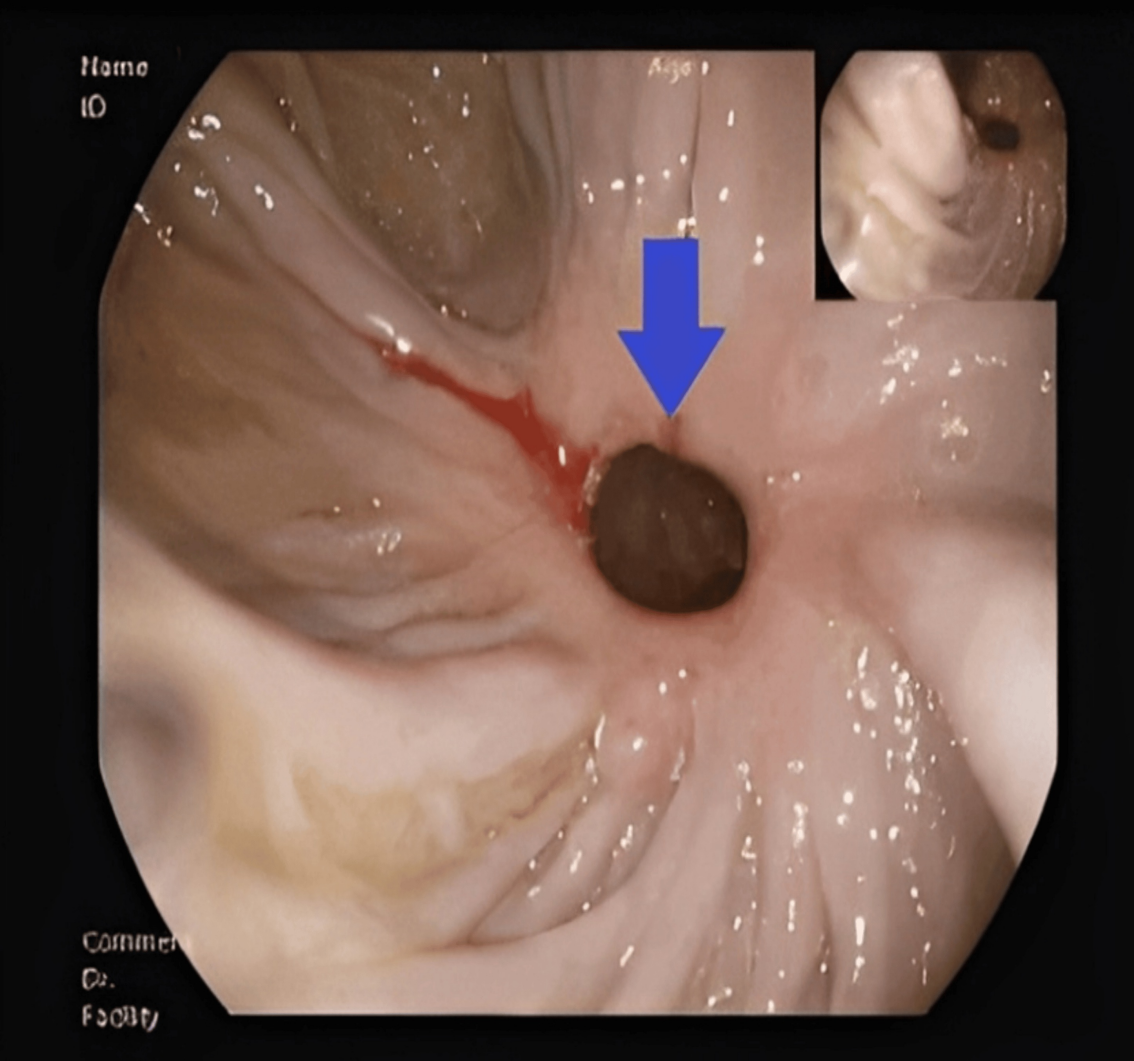
Endoscopic Follow-Up Demonstrating Closure Failure Upper endoscopy six weeks after over-the-scope clips (OTSC) placement, showing a persistent duodenocolic fistula (blue arrow). Minor digital enhancement applied.

Two endoscopic closure attempts using OTSC were performed (Figure 4). However, follow-up endoscopy six weeks later demonstrated persistent fistula patency (Figure 5).

**Figure 6:**
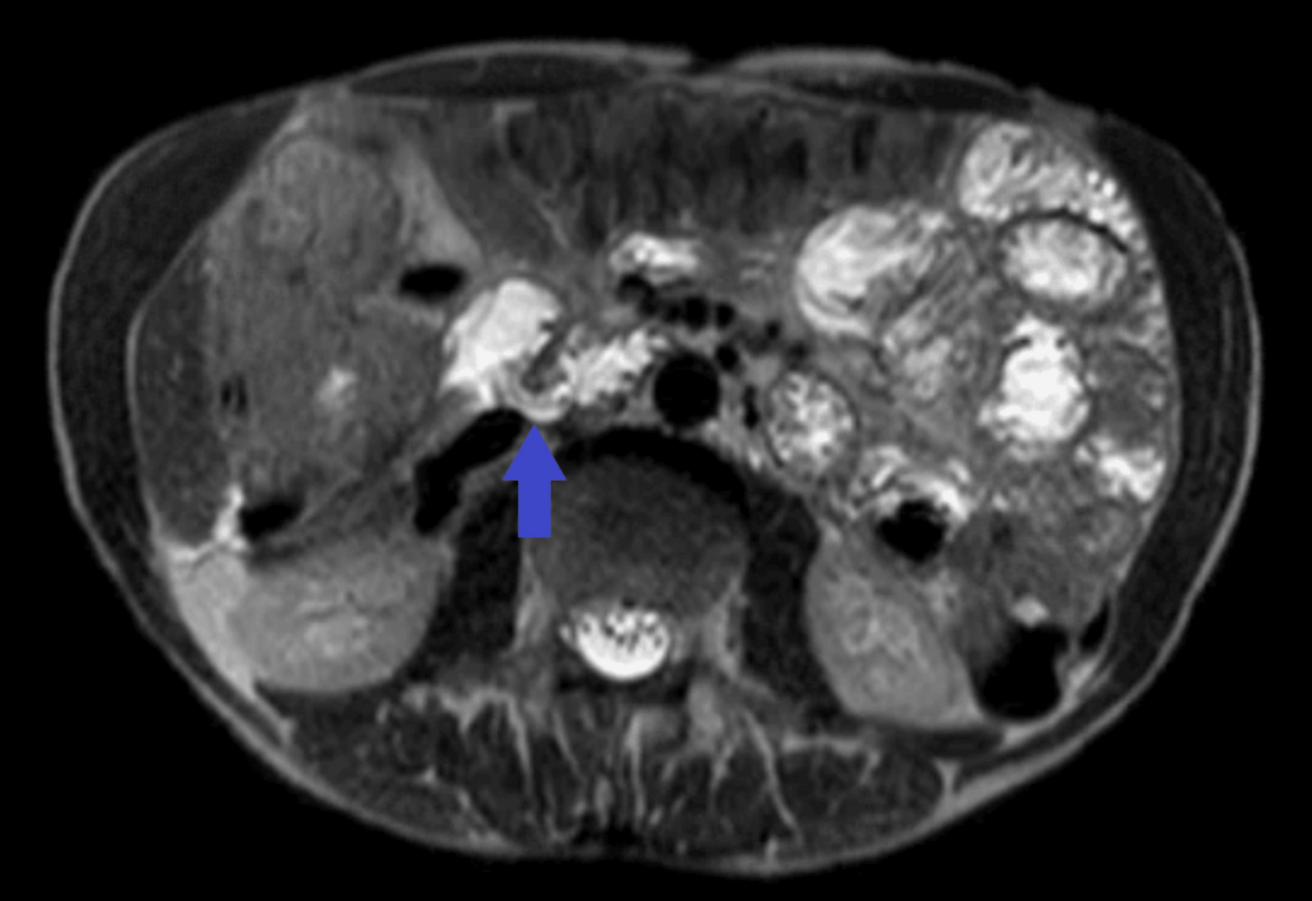
MR Enterography Demonstrating the Fistulous Tract T2-weighted magnetic resonance (MR) enterography image demonstrating the fistulous tract (blue arrow), with fluid content passing from the duodenum to the hepatic flexure of the colon.

Magnetic resonance (MR) enterography was subsequently performed to further characterize the fistulous tract and definitively exclude underlying inflammatory bowel disease or malignancy, confirming the presence of the fistula (Figure 6).

**Figure 7:**
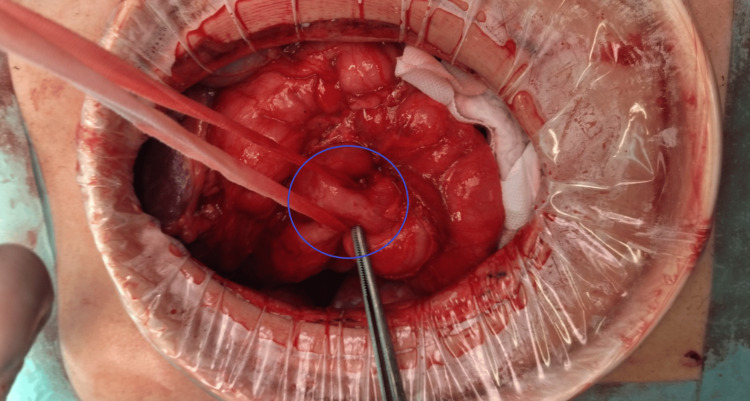
Intraoperative Identification of the Duodenocolic Fistula Intraoperative image showing the fistulous tract (circled), located between the D2–D3 transition and the hepatic flexure. The tract was isolated using umbilical tape to facilitate safe dissection.

**Figure 8:**
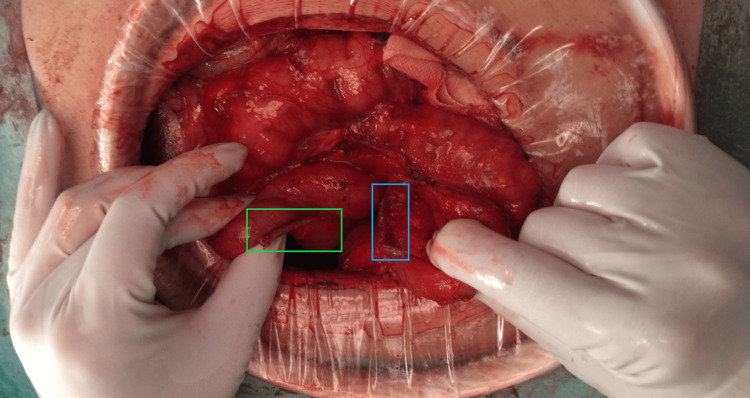
Primary Surgical Closure After Using a Linear Stapler Final intraoperative view after primary closure of the duodenocolic fistula. The stapler line (linear gastrointestinal anastomosis (GIA) 55 mm, blue load) securely seals both sides of the fistula: the duodenal stump (blue rectangle) and the colonic stump (green rectangle).

Following the failure of two endoscopic closure attempts using OTSC and progressive malnutrition, surgical intervention was planned. The patient underwent five weeks of preoperative nutritional optimization, during which serum albumin increased from 2.2 g/dL to 3.4 g/dL. Surgical exploration via a supra-umbilical midline incision revealed a 1-cm duodenocolic fistula at the D2-D3 transition and the hepatic flexure of the colon. Primary closure using a linear cutting stapler was performed to ensure a secure and efficient repair while minimizing tissue manipulation and tension on the suture line, without intraoperative complications (Figures 7, 8).

Histopathology showed nonspecific chronic inflammation.

Postoperatively, the patient resumed a liquid diet on postoperative day two and was discharged on day four with normal bowel function. At the one-year follow-up, diarrhea had resolved completely, BMI had increased to 18.8 kg/m², and laboratory abnormalities had normalized, with a marked improvement in quality of life.

## Discussion

Duodenocolic fistulas are uncommon clinical entities, with the majority of cases resulting from Crohn's disease, malignancy, or postoperative complications [1,3]. Idiopathic presentations, such as in the present case, are exceedingly rare and pose a significant diagnostic challenge. Patients typically present with chronic diarrhea, severe weight loss, anemia, and protein-calorie malnutrition, consistent with the clinical picture observed in our patient [2].

Accurate diagnosis relies on radiologic evaluation. Water-soluble contrast studies are particularly effective in demonstrating direct communication between the duodenum and the colon [4]. Cross-sectional imaging modalities such as CT and MR enterography provide additional anatomical detail and help exclude inflammatory or neoplastic etiologies [5,6]. Endoscopic treatment with OTSC is increasingly reported for gastrointestinal fistulas; however, outcomes vary substantially depending on fistula size, chronicity, and the presence of mature epithelialized tracts [7,8].

In the present case, two attempts at OTSC closure were unsuccessful, likely reflecting the chronic nature and 1-cm size of the fistula, which may have limited the effectiveness of endoscopic therapy. These observations underscore that endoscopic approaches must be carefully considered on a case-by-case basis, as surgical repair remains the gold standard for definitive management when they fail or are deemed unsuitable [3,8]. The operative strategy in this case, primary stapled closure of both the duodenal and colonic openings, was considered appropriate given the benign, idiopathic etiology and the absence of active inflammation or malignancy. This approach allowed for a secure and tension-free repair while avoiding more extensive procedures, such as segmental resection or anastomosis. Preoperative nutritional optimization was an essential component of care, given the high prevalence of malnutrition in these patients [2]. Long-term outcomes following surgical correction are generally favorable, with reported resolution of diarrhea and progressive weight gain [8]. At the one-year follow-up, the patient demonstrated complete symptom resolution and substantial recovery in nutritional status. Therefore, while endoscopy represents a valuable diagnostic and potentially therapeutic tool, surgeons should maintain a low threshold for timely operative intervention in idiopathic duodenocolic fistula, particularly in the presence of significant malnutrition or features of a chronic, mature fistulous tract.

## Conclusions

Idiopathic duodenocolic fistula is an exceptionally rare condition that should be considered in adults presenting with chronic diarrhea, malabsorption, and unexplained weight loss. When endoscopic closure fails, surgical repair remains the most effective definitive treatment, offering durable symptom resolution and recovery of nutritional status. This case highlights the importance of timely diagnosis, careful perioperative optimization, and a tailored surgical strategy to achieve optimal patient outcomes.
